# Herb induced liver injury after using herbal medicine: A systemic review and case-control study

**DOI:** 10.1097/MD.0000000000014992

**Published:** 2019-03-15

**Authors:** Nai-Hui Lin, Hsiu-Wu Yang, Yu-Jang Su, Chen-Wang Chang

**Affiliations:** a Emergency Department and Poison Center, Mackay Memorial Hospital, Taipei City; b Department of Medicine, Mackay Medical College, New Taipei City; c Department of Oral Hygiene, College of Oral Medicine. Taipei Medical University, Taipei; d Yuanpei University of Medical Technology, Hsinchu; e Division of Gastroenterology, Department of Internal Medicine, Mackay Memorial Hospital; f MacKay Junior College of Medicine, Nursing and Management, Taipei, Taiwan.

**Keywords:** herb induced liver injury, herbal and dietary supplements (HDS), herbal medicine

## Abstract

In Taiwan, traditional herbal medication was included in Taiwan's National Health Insurance (NHI) system since 1996 and in 9 out of 10 hospitals have developed their own departments of traditional medicine. This study aims to address the herb-induced liver injury (HILI) after using herbal medicine on the relationship between age, gender, epidemiology, laboratory data, pathogenesis, mobility, and mortality.

We searched the PubMed database with “hepatitis after herbal medicine” and “in human” till 2018 April and returned 163 articles in a systemic review manner. Two cases reports describing in-vitro liver injury were excluded. Reviews and articles without the detailed report, laboratory data and history were excluded from this study. In the end, there were 53 articles enrolled in this study. These enrolled literatures are from France (n = 13), Germany (n = 12), Switzerland (n = 5) United States of America (n = 4), Korea (n = 4), Hong Kong (n = 4), Greece (n = 3), China (n = 2), Canada (n = 1), Italy (n = 1), Thailand (n = 1), Finland (n = 1), Taiwan (n = 1), and Japan (n = 1). The data were analyzed with a commercial statistical software Stata/SE 12.0 program Stata Corporation, College Station, TX, USA. Statistical χ^2^ tests were performed and the significance was set at a *P* value of less than .05 (2-tailed).

The ages are ranged from 15 to 78 years with the mean ± SD (standard deviation) of 48.3 ± 16.2 years old. The majority of cases are female (n = 37). In elderly, man is more commonly seen than female in HILI (37.5% vs 10.5%, *P* = .02). Female is vulnerable to cholestatic type of HILI than male (21.1% vs 0.0%, *P* = .04). Of all the cases in HILI, using pure substance are more commonly seen than mixed substance (*P* = .02). In gender, male patients have higher alanine aminotransferase (GPT) (IU/L) level in HILI than female ones (1560 ± 819 vs 1047 ± 706, *P* = .03).

In HILI, the female is more commonly seen than male, but less than male in the elderly. The pure substance more often happens to HILI than mixture substance. Female is predominant in the cholestatic type of HILI. The major prevalence of HILI is in Europe rather than Asia. HILI cases in Europe is 2.75-fold than in Asia. This could be due to fewer reports of the herb induced liver injury in Asia compared to Europe. Prevention of HILI is the best policy, because it needs to take 78 ± 59 days to recover.

## 1 Introduction

People promote their health and make herbal and dietary supplements (HDS) become more and more popular around the world nowadays. People taking HDS is thought to believe that it is more safe, effective and without any complication for their long-term health.^[1]^ However, Navarro et al had reported several cases of hepatotoxicity after usage of HDS.^[2]^ In Taiwan, traditional herbal medication (TCM) was included in Taiwan's National Health Insurance (NHI) system since 1996 and in 9 out of 10 hospitals have developed their own departments of traditional medicine.^[3]^ Teschke et al had described a review of TCM causing liver Injury implying herbs may contain extensive venomous molecules, accumulation of hazardous components through the intestinal process and hepatic systems in 2014.^[4]^ It is very difficult for clinical physicians in identifying and early diagnosing the patients with herb-induced liver injury (HILI) by history taking and physical examination.^[5]^ Besides, patients seldom informed the family physicians of using these herbs because they can easily get herbs or complementary medications everywhere without doctor's approved or prescriptions.^[6]^

HILI presents in variable unspecific signs and symptoms with general weakness, gastrointestinal (GI) symptoms (i.e., abdomen distension, abdomen pain or fullness), or typical hepatitis symptoms. Presentations are very similar to drug-induced liver injury (DILI) or at least share the same pictures of liver injury which makes the differential diagnosis more difficult.^[5]^ There are many causality assessments helpful to highlight the possibility of HILI. Teschke et al claimed council for international organizations of medical sciences (CIOMS) scale is liver-specific, quantitative for liver and validated for hepatotoxicity but not suitable for non-hepatic adverse drug reactions (ADRs). In current causality assessment methods, only Naranjo scale is validated for non-hepatic ADRs.^[7]^

Most herbal-induced hepatotoxicity cannot reproduce in experimental animal models. Thus, it is difficult to understand the pathogenetic factors.^[5]^ Current medical technology still cannot provide a highly specific and high sensitivity marker or standardized measurements to make a definite diagnosis of HILI. Clinical physicians can only make the diagnosis of HILI by exclusion. De Boer et al stated diagnosis of some hepatotoxicities after specific herbal are made according to the typical clinical presentations.^[5,6]^ This study aims to address the hepatitis after using herbal medicine (HILI) on the relationship between age, gender, in herbal toxicity, the typical clinical pictures, epidemiology, laboratory data, pathogenesis, mobility, and mortality.

## 2 Materials and methods

**Figure 1:**
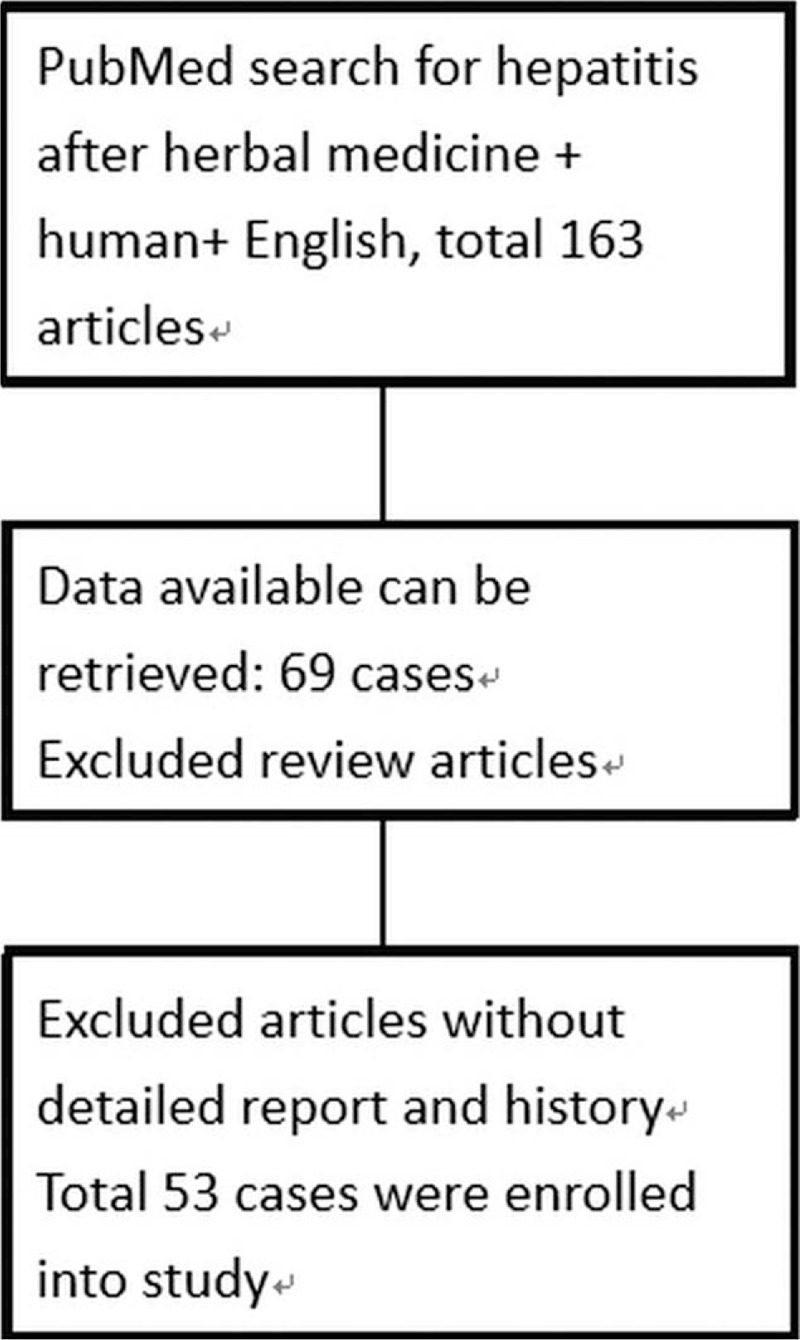
The process of enrollment 53 hepatitis after using herbal medicine (HILI). HILI = herb-induced liver injury.

A systemic review was designed to investigate the etiology, epidemiology, mechanism, recovery days of liver injury after using herbal medication. A search of “hepatitis after herbal medicine” and “in human” till 2018 April from the PubMed database returned a total of 163 articles. Excluding the cases, reports of liver injury were in vitro. Reviews and articles without detailed report, laboratory data and history were excluded from this study. In the end, there were 53 articles enrolled in this study. The process of enrollment is shown in Figure 1. The combined data and descriptions of herbal medicine related hepatitis all over the world is shown in Table 1. We collected information of year happened, country, age (years old), gender, herbal substance, Glutamic oxaloacetic transaminase (GOT) (IU/L), alanine aminotransferase (GPT) (IU/L), total and direct bilirubin (mg/dl), days to recover (days), and mortality or not, and analyzed systemically.

**Table 1:**
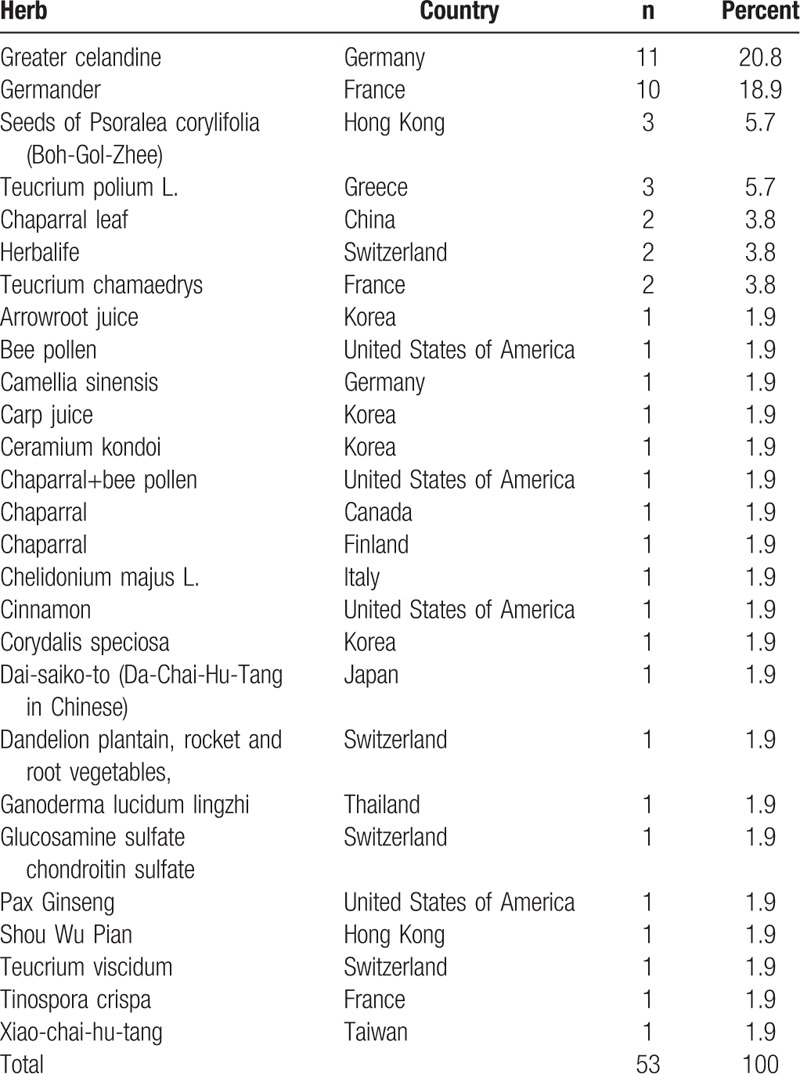
List of herbal medicine related hepatitis all over the world.

### 2.1 Classification of herbal hepatotoxicity

Liver injury was defined by alanine aminotransferase (GPT), alkaline phosphatase (ALP) and R ratio equal to the ratio between GPT and ALP relative their respective upper limits of normal ranges (ULN), and classified as below

1. Hepatocellular type injury: if R ratio ≥ 5 and GPT ≥ 2 × ULNGPT
2. Cholestatic type injury: if R ratio ≥ 2 and ALP ≥ 2 × ULNALP
3. Mixed type injury: if 2 < R < 5 and both GPT ≥ 3ULNGPT and ALP ≥ 2 × ULNALP.

These enrolled literature are from France (n = 13), Germany (n = 12), Switzerland (n = 5) United States of America (n = 4), Korea (n = 4), Hong Kong (n = 4), Greece (n = 3), China (n = 2), Canada (n = 1), Italy (n = 1), Thailand (n = 1), Finland (n = 1), Taiwan (n = 1), and Japan(n = 1).

### 2.2 Statistical analysis

The data were analyzed with a commercial statistical software Stata/SE 12.0 program Stata Corporation, College Station, TX. Statistical χ^2^ tests were performed and the significance was set at a *P* value of less than .05 (2-tailed).

## 3 Results

**Figure 2:**
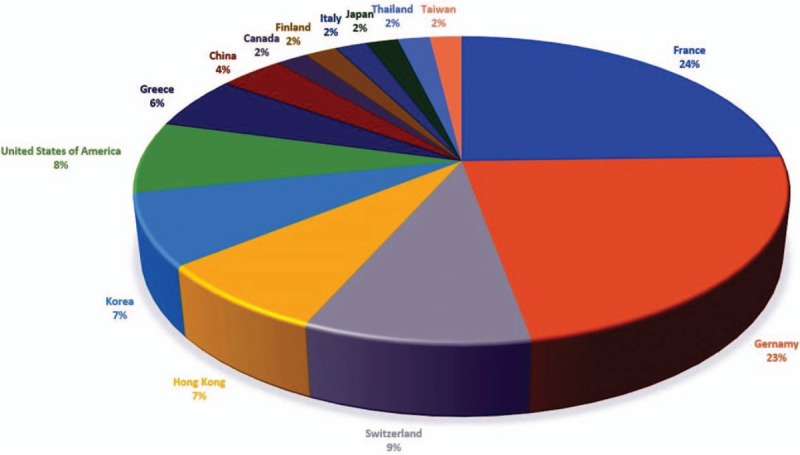
List of herbal substance and countries in HILI. HILI = herb-induced liver injury.

The 53 cases last from the year 1986 to 2016. The majority of cases are reported in France (24%) and Germany (23%), followed by Switzerland (9%), the United States of America (8%), Hong Kong (7%), Korea (7%), and Greece (6%). There are also cases report in China, Canada, Italy, Thailand, Finland, Taiwan, and Japan (Fig. 2).

The most commonly reported herbal medicine related hepatitis are Greater celandine reported in Germany (20.8%) and Germander reported in France (18.9%). The other contributing herbal medicine also include Seeds of *Psoralea corylifolia* (Boh-Gol-Zhee), Teucrium polium L., Chaparral leaf, Herbalife, *Teucrium chamaedrys*, Arrowroot juice, Bee pollen, *Camellia sinensis*, Carp juice, *Ceramium kondoi*, Chelidonium majus L, Cinnamon, *Corydalis speciosa*, Dai-saiko-to (Da-Chai-Hu-Tang in Chinese), Dandelion plantain, rocket and root vegetables, Ganoderma lucidum lingzhi, Glucosamine sulfate, chondroitin sulfate, Pax Ginseng, Shou Wu Pian, Teucrium viscidum, Tinospora crispa, and Xiao-chai-hu-tang (Table 1).

**Figure 3:**
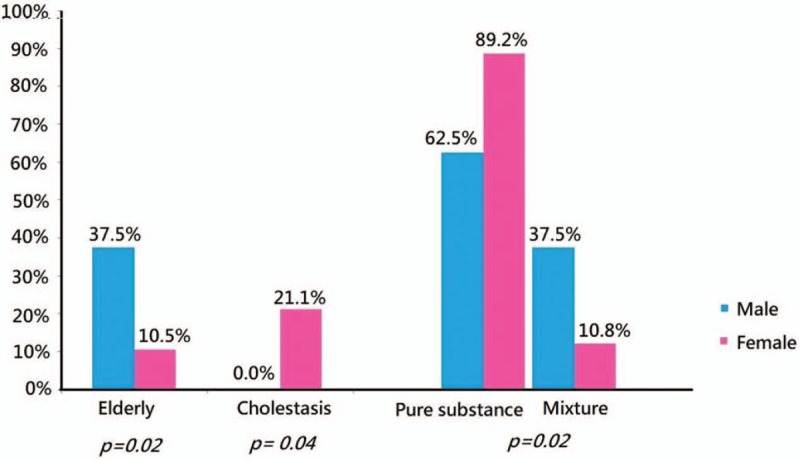
Comparisons in gender with statistical difference in elderly, cholestasis, and herbal substances.

The ages are ranged from 15 to 78 years with the mean ± SD (standard deviation) of 48.3 ± 16.2 years old. The majority of cases are female (n = 37). In elderly, man is more commonly seen than female in HILI (37.5% vs 10.5%, *P* = .02). Female is vulnerable to cholestatic type of HILI than male (21.1% vs 0.0%, *P* = .04). Of all the cases in HILI, using pure substance are more commonly seen than mixed substance (*P* = .02). In gender, male patients have higher alanine aminotransferase (GPT) (IU/L) level in HILI than female ones (1560 ± 819 vs 1047 ± 706, *P* = .03) (Table 2 and Fig. 3).

**Table 2:**
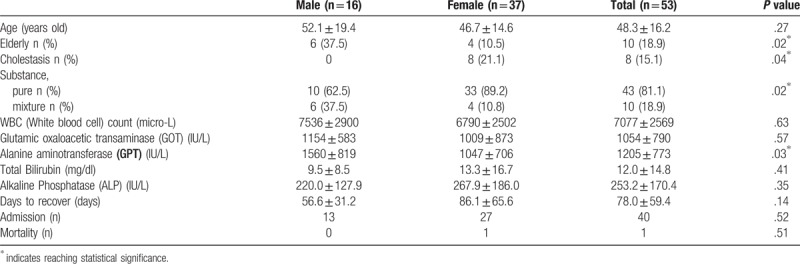
Gender comparisons in hepatitis after herbal medicine.

There is no statistical significance in white blood cell count (micro-L), glutamic oxaloacetic transaminase (IU/L), total bilirubin (mg/dl), alkaline phosphatase (IU/L), days to recover, and mortality in gender comparisons in hepatitis after herbal medicine (Table 2).

## 4 Discussion

### 4.1 Age and gender

There are many theories about the pathogenesis of liver injury after using herbal medicine. Raschi et al have divided the suspicious pathogenesis into three main related factors: host, drug, and environments.^[8]^ A similar concept in DILI, Robert et al implicated the similar pathogenesis of idiosyncratic DILI, included age, gender, genetic, drug-drug interaction, drug dosage, and the environmental factors.^[9]^ In the host factors, we find in the elderly group, the male is more vulnerable to herbal toxicity than female. As in DILI, the male is tent to overreacted in specific drugs such as anti-viral drug and azathioprine than women.^[8,10]^ However, some reviews described the different opinions, the older female tent to get liver injury after using non-body-building HDS.^[11,12]^ Rolf Teschke et al also reported the female is the predominates gender in HILI and in a Korea study, all the cases above 50 years old identified with HILI were all females.^[7,13]^ Females are believed to sensitive to specific drugs as specific antibiotics and some antipsychotics agents.^[14]^ Shapiro et al also reported the elderly and the women are more commonly seen in liver injury caused by any medication.^[15]^ In our study, female accounted for 69.8% cases of HILI, but in the elderly, male is predominant in HILI. Considering in the old age, gender may have different susceptibility in HILI which need more studies in the aging population in the future.^[9]^

### 4.2 Prevalence

TCM has many alternative names such as complementary and alternative medicines (CAMs), traditional Asian medicine (TAM) or traditional oriental medicine (TOM), and Kampo Medicine in Japan. CAMs, focusing on herbal usage, is a very popular traditional medicine in China for over thousands of years, though its treatment efficacy cannot be proven. TAM is still a very popular medication and becomes more and more popular in years worldwide. We surprisingly find there are only 2 cases reported in HILI of China were included in our study. Yuan Zhoua et al had announced a Chinese literature research for DILI and HILI, revealed the TCM related liver injury often classified into the “drug” induced liver injury, not HILI.^[16]^ Yuan Zhoua et al also considered the epidemiology of Chinese medicine related liver injury in China, not being well recognized.^[16]^ A review article written by Melchart et al, among 1507 patients who received TAM, only 2 patients were recognized symptomatic.^[17]^ In Asian countries, such as China and Korea, the etiology in HILI and DILI, may not be well described, resulting in relatively lower frequency in China, and may not meet the global classification of liver injury type as used in Europe or USA, where showed progression in the usage of HDS over the last 10 years.^[18–20]^ For the reason above, Gaby Danan et al strongly recommend China to report HILI cases with precise assessment as (Roussel Uclaf Causality Assessment Method) RUCAM and should exclude all the possibility that might cause liver injury.^[21]^

Nowadays, there are many studies in genome-wide association (GWA) approaching genetic pathogenesis in HILI and DILI.^[7]^ Daly AK et al discovered the human leukocyte antigen (HLA) gene is strongly associated with DILI which caused by flucloxacillin.^[22]^ HLA-B^∗^5701, an immune-related gene, consider playing an important hypersensitivity-mediator in the overreaction to abacavir, resulting in severe adverse drug reactions as acute liver failure, which prevalence in Europe.^[7,23]^ This is compatible with our study discovered the major prevalence of HILI in Europe rather than Asia. In our study, HILI cases in Europe are 2.75-fold Asia (66% vs 24%).

### 4.3 Classification into hepatocellular, cholestatic injury, and mixed type

Similar to DILI, Teschke R et al consider the mechanism of HILI can be classified into idiosyncratic and intrinsic injury. Idiosyncratic injury, which dosage independent, can further subclass into metabolic type and immunologic type.^[24]^ For example, the top 2 herbals account for almost 40% of HILI cases in our study are Greater celandine (n = 11, 20.8%) and Germander (n = 10, 18.9%), both classified in the metabolic type of idiosyncratic injury, due to their liver-toxicity metabolites. But Benninger et al had done a case series for hepatitis after Greater celandine ingested and consider the greater celandine may also have immunologic toxicity due to the titer of antibodies.^[25]^ It is very cumbersome and complicated process of making herbal and other supplements. And the possibility of toxicity in herbal included mistaking the wrong herbs, not appropriate collections, not suitable manipulation, poor preservation, inappropriate extraction may associate with herbal toxicity.^[26]^ Although most of the herbals toxicity remains unclear, we can assess the herbal toxicity through lab criteria which included alanine aminotransferase (GPT), alkaline phosphatase (ALP) and the ratio between GPT to ALP relative their respective upper limits of normal ranges (ULN).^[12]^ Hepatocellular injury is the dominant liver injury type in herbal toxicity which meets the below criteria as^[1]^ GPT 3 times above ULN of GPT^[2]^ R ratio over or equal to 5. The cholestatic injury is defined as^[1]^ ALP 2 times ULN of ALP^[2]^ R ratio <2.^[12,24,27]^ According to our research, we find the women is the predominant gender in cholestatic injury after herbal usage than men (*P* < .05). In Björnsson et al reported several drug-induced liver diseases, the female is accounted for 59% of the cholestatic types.^[14]^ Russmann et al also found Flucloxacillin associated cholestatic type DILI is most seen in women.^[28]^ But the study in the USA showed no significant gender difference in cholestatic type of DILI.^[29]^

HILI has similar mechanisms and symptoms with DILI. They share almost identical clinical, biochemical, and histological features of hepatotoxicity.^[30]^ Conventional synthetic drugs usually have well-established liver injury mechanisms and identified compounds that involved. Classification of herbal hepatotoxicity is challenging to define because the herbal product is often a combination of various constituents and involves various confounding variables.^[31]^

HILI can be broadly categorized as intrinsic vs idiosyncratic, and the latter further classified into allergic vs non-allergic.^[32]^ Intrinsic hepatotoxicity is dose-dependent and predictable above specific thresholds. The intrinsic toxicity of the herbs is dosage causing severity of liver injury. In contrast, idiosyncratic hepatotoxicity is usually unpredictable happened without dose-related respond. Allergic idiosyncratic hepatotoxicity involves adaptive immune reactions with the presence of typical symptoms such as fever, skin reactions, eosinophilia, and the formation of autoantibodies, and a short latency period in particular after re-challenge.^[32]^ The risk of acute liver failure associated with idiosyncratic hepatotoxicity is usually less than 1 per 10,000 patients.^[32]^

The pathophysiologic mechanism of drug-induced hepatotoxicity can be divided into hepatocellular and extracellular processes.^[32–34]^ Hepatocellular process start from covalent binding of a substance to intracellular proteins results in a decrease in adenosine triphosphate (ATP) levels and actin disruption, cause cell swollen and rupture. If substance covalent to cytochrome P450 enzyme results in immunogens activating T-cells and cytokines formation, and evoking consequence immune response. Tumor necrosis factor (TNF) activation apoptotic pathways to hepatocytes results in programmed cell death. Extracellular processes include disruption of the transport proteins cause interruption of transport systems, result in the impairment of canalicular transport of bile salts and cholestasis. Besides, toxic metabolites excreted in bile may further destruction bile duct epithelium. Hepatic biochemistry pattern of HILI can be categorized into three types: hepatocellular, cholestatic, or mixed pattern.^[2]^ Hepatocellular type represents hepatotoxicity are mainly hepatocellular process, while cholestatic type are extracellular process, and mixed type are the combination of 2 processes.

### 4.4 Significance of results

Our study collects 53 cases with pure or mixed substances herbal product, the 3 most frequently reported hepatotoxic herbal products are greater celandine (n = 12, 22.64%), germander (n = 12, 22.64%), and chaparral (n = 5, 9.43%). We discuss them as below.

#### 4.4.1 Greater celandine

Greater celandine (Chelidonium majus L.) is a plant of the poppy family that grows wildness in Asia and Europe.^[35]^ It has been used for centuries to treat gastrointestinal complaints, dyspepsia, and gallbladder disease. Several cases of liver injury associated with greater celandine have been reported, mostly from Germany.^[35–40]^ Lobular and portal inflammation with bridging fibrosis and eosinophilic infiltrates were observed in most of the liver histology.^[25]^ More than 20 different kinds of alkaloids can be found in greater celandine, including chelerythrine, sanguinarine, berberine, chelidonine, and coptisine.^[40]^ However, hepatotoxicity cannot be found by each of them. Because there is no evidence of dose dependency, and with long and variable latency as well as the presence of eosinophilic infiltrate, hepatotoxicity related to patient's idiosyncrasy also considered by investigators.^[25,41]^

#### 4.4.2 Germanders

Germanders (Teucrium chamaedrys L.) are found in Europe and the Middle East, and the aerial parts of the plant are used.^[42]^ Germander has been used for centuries as traditional herbal remedies for their diuretic, diaphoretic, antipyretic, antispasmodic, anti-inflammatory, antihypertensive, anorexic, analgesic, hypoglycemic, and hypolipidemic properties.^[43,44]^ Germander has become popular as a remedy for weight loss in the past few decades.^[45]^ In 1986, germander-containing capsules and tea preparation were approved in France as an adjuvant to weight control. But after several advert incident germander-associated acute, chronic, and even fulminant hepatitis, the remedy was withdrawal from the market and banning it in 1992.^[46,47]^ In general, germander-induced hepatotoxicity may occur after approximately 2 to 3 months of ingestion at the manufacturer's recommended doses (600–1600 mg/ day). Symptoms related to hepatotoxicity are non-specific and typically include fatigue, nausea, and the development of jaundice associated with marked elevation of serum aminotransferase levels,^[45–49]^ the typical features of hepatocellular adverse reactions.

#### 4.4.3 Chaparral

Chaparral (Larrea tridentata), commonly known as creosote bush or greasewood, is a botanical dietary and “energy” supplement. It is prepared from the leaves of the evergreen desert shrub that can be found in the southwestern U.S. and Mexico.^[50]^ Native Americans have traditionally used it medicinally for the treatment of various ailments including respiratory tract infections, rheumatic pain, abdominal pain, chicken pox, and snakebite pain.^[51]^ Currently, chaparral has been marketed in the form of tea, tablets, capsules, and salves (for burns), weight loss, liver tonic, blood purifiers, and treatment of skin disorders.^[51]^ In the 1990s, a series of incidents regarding chaparral-associated hepatotoxicity were reported to the FDA.^[51]^ The hepatotoxicity of chaparral is generally attributed to nordihydroguaiaretic acid (NDGA). NDGA is an antioxidant and was once used as a preservative in foods for humans and for pharmaceuticals. NDGA can inhibit lipoxygenase and cyclooxygenase,^[52,53]^ and hence, it affects many intrahepatic pathways. The predominant pattern of liver damage was cholestatic hepatitis with high serum transaminases and the elevation of bilirubin and alkaline phosphatase.^[54]^ Liver injuries ranging from mild hepatitis to cirrhosis and fulminant liver failure were reported.^[51]^

### 4.5 Laboratory tests

In total bilirubin value, there is no significant statistical difference between the 2 gender groups. Alanine aminotransferase (GPT) is a primary indicator for hepatocyte damage severity in biochemistry, and in our study, male victims have higher average GPT then female (1560 ± 819 vs 1047 ± 706, *P* = .03). Due to the male group has a higher portion of elderly (37.5% vs 10.5%, *P* = .02), we consider the result represent elderly people are generally at a higher risk for HILI or DILI. According to other authors hypothesis, old age patients have decreased hepatic and renal clearance, higher probability of drug-to-drug interactions, reduced hepatic blood flow, variation in serum drug binding, and lower hepatic volume.^[55]^ Eight patients were classified as cholestasis and mixed type hepatotoxicity, and all of them are female. A report in 2009 from Spain described that older age is a determinant for cholestatic damage with a male predominance, and cytolytic damage is associated with younger female.^[56]^

### 4.6 Days to recover and outcome

Patients who recover from herbal-induced hepatotoxicity generally have a favorable prognosis. Average days to recover from HILI in our study is 78.0 ± 59.4 days, without reaching statistical difference between genders (56.6 ± 31.2 vs 86.1 ± 65.6, *P* = .14). Most of the patients (n = 40) need to admission for supportive care, with a similar proportion in two groups (13 vs 27, *P* = .52). Only one female case in our study suffered acute liver failure and mortality.

### 4.7 Prevention

Although the hepatotoxic potential of herbal products has been recognized for years, there is a definite lack of reliable population-based epidemiological studies specifically relating to the incidence of herbal hepatotoxicity. Herbals are considered by food and dietary supplement and had a lower threshold of required evidence for safety.^[57]^ In the U.S., the Dietary Supplement Health and Education Act (DSHEA) of 1994 was an amendment to the US Federal Food, Drug, and Cosmetic Act (FD&C).^[2]^ The DSHEA requires that manufacturers establish product safety before marketing. Manufacturers must ensure that the product label is truthful and not misleading. The label must contain a complete list of all ingredients contained in the product and the identity of the manufacturer. Herbal products that were already in distribution prior to 1994 are allowed on the market, the safety can only depend on user experience without scientific evidence. For these reasons, the National Library of Medicine and the DILI Network study group has established the LiverTox website.^[58]^ This online database contains more than 1000 products that have been implicated DILI with comprehensive clinical information for both the general physician and the specialist. However, we believe plenty amount of HILI incidents were never reported due to causality assessment is challenging. Clinicians should keep in mind to take a history of herbal products to use while evaluating a patient with abnormal liver function.

## 5 Conclusion

Herbal and dietary supplements are popular all over the world, and herbal medicine usages are not only Asian countries but also Europe and Americans. In hepatitis after using herbal medicine (HILI), the female is more commonly seen than male, but less than male in the elderly. Pure substance more often happens to HILI than mixture substance. Female is predominant in the cholestatic type of HILI. The major prevalence of HILI is in Europe rather than in Asia. HILI cases in Europe is 2.75-fold than in Asia. This could be due to fewer reports of the herb induced liver injury in Asia compared to Europe. Prevention of HILI is the best policy because it needs to take 78 ± 59 days to recover.

## 6 Limitation

There are some confounding factors in this study. Because it is based on the PubMed search, and possibly there are some HILI cases unreported all over the world. Thus, it could be underestimated in number of HILI cases. After all, it is a retrospective study; some data losses are inevitable to influence the statistical result.

## Author contributions

**Conceptualization:** Yu-Jang Su

**Data curation:** Nai-Hui Lin, Hsiu-Wu Yang, Yu-Jang Su, and Chen-Wang Chang

**Formal analysis:** Yu-Jang Su and Chen-Wang Chang

**Investigation:** Nai-Hui Lin, Hsiu-Wu Yang, and Yu-Jang Su

**Methodology:** Nai-Hui Lin, Hsiu-Wu Yang and Yu-Jang Su, and Chen-Wang Chang.

**Project administration:** Yu-Jang Su

**Resources:** Nai-Hui Lin, Hsiu-Wu Yang

**Supervision:** Yu-Jang Su

**Writing – original draft:** Nai-Hui Lin, Hsiu-Wu Yang, and Yu-Jang Su

**Writing – review & editing:** Yu-Jang Su

Yu-Jang Su orcid: 0000-0003-0218-1944.
